# The hypoxia signalling pathway in haematological malignancies

**DOI:** 10.18632/oncotarget.15981

**Published:** 2017-03-07

**Authors:** Marta Irigoyen, Juan Carlos García-Ruiz, Edurne Berra

**Affiliations:** ^1^ Centro de Investigación Cooperativa en Biociencias CIC bioGUNE, Derio, Spain; ^2^ Servicio de Hematología y Hemoterapia, BioCruces Health Research Institute, Hospital Universitario Cruces, Barakaldo, Spain

**Keywords:** hypoxia, hypoxia-inducible factors, haematological cancers, cancer stem cells, resistance

## Abstract

Haematological malignancies are tumours that affect the haematopoietic and the lymphatic systems. Despite the huge efforts to eradicate these tumours, the percentage of patients suffering resistance to therapies and relapse still remains significant. The tumour environment favours drug resistance of cancer cells, and particularly of cancer stem/initiating cells. Hypoxia promotes aggressiveness, metastatic spread and relapse in most of the solid tumours. Furthermore, hypoxia is associated with worse prognosis and resistance to conventional treatments through activation of the hypoxia-inducible factors. Haematological malignancies are not considered solid tumours, and therefore, the role of hypoxia in these diseases was initially presumed to be inconsequential. However, hypoxia is a hallmark of the haematopoietic niche. Here, we will review the current understanding of the role of both hypoxia and hypoxia-inducible factors in different haematological tumours.

## HAEMATOLOGICAL MALIGNANCIES

Haematological malignancies (HMs) are tumours characterized by uncontrolled proliferation of cells from the immune and the haematopoietic system. In Europe, recent analyses show an age-standardized incidence rate of 32 tumours per 100.000 persons/year [1]. These heterogeneous pathologies are nowadays classified based on the affected cell lineage as myeloid or lymphoid. Neoplasias of lymphoid origin are the most frequently observed (75% of the total HMs) with multiple myeloma (MM), small B-cell lymphocytic lymphoma (SBLL) / chronic lymphatic leukaemia (CLL), diffuse large B-cell lymphoma (DLBCL) and Hodgkin lymphoma (HL) being the most common. Acute myeloid leukaemia (AML), myeloproliferative neoplasms (MPN) and myelodysplastic syndromes (MDS) account for the highest rate among the myeloid malignancies [2, 3]. Despite the differences in biology, clinical manifestations and outcome between disease subtypes, an overall significant progress in terms of diagnosis and cure rates has been achieved in the past decades. To date, the majority of paediatric acute lymphatic leukaemia (ALL) and chronic myeloid leukaemia (CML) cases are indeed cured or well controlled. Overall survival at 5 years is over 60% and around 80% in patients with non-Hodgkin and Hodgkin lymphoma, respectively [4, 5]. In contrast, CLL and AML have a high risk of relapse, and MM remains mostly incurable [6, 7, 8]. There is compelling evidence that a small population of stem-like cancer cells with the capacity for self-renewal and differentiation accounts for resistance and recurrence in many types of cancer [8, 9]. In the context of HMs, these cells are known as leukaemia-, lymphoma- or myeloma-initiating cells (from now on referred to as haematological cancer stem cells, HCSCs). Although there is some controversy on the phenotype of such a population(s), HCSCs are certainly involved in the initiation and maintenance of HMs [10–14].

**Figure 1:**
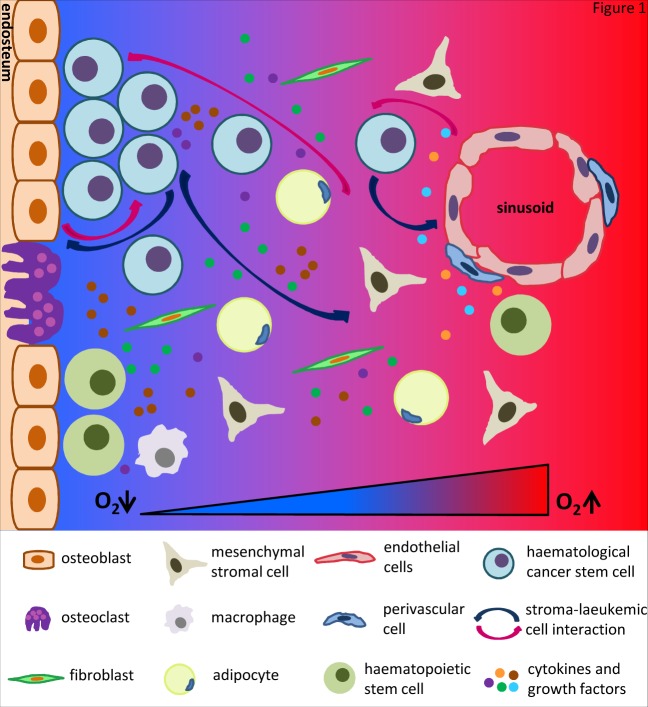
Picture of normal haematopoietic stem cells (HSCs) and haematological cancer stem /initiating cells (HCSCs) niches in the bone marrow Both HSCs and HCSCs niches are composed of a collection of different cell types, growth factors and cytokines, which are localized close to the endosteum and sinusoids. Oxygen levels decrease from the sinusoids to the endosteum. HCSCs expand within the hypoxic endosteal niche while take advantage of the vascular niche to colonize distant organs.

Cancer research has been mostly focused on cancer cells themselves. However, it is now well accepted that tumours are complex tissues sustained by the dynamic interactions between cancer cells and their environment. This environment consists of a number of cell types (fibroblasts, endothelial cells, adipocytes, macrophages, antigen-presenting cells, etc) and many different molecules (growth factors, cytokines, chemokines, extracellular matrix, adhesion molecules, etc), which are commonly referred to as the tumour stroma [15]. Forty years ago, studies performed in the laboratory of Dr John Trentin demonstrated that stromal cells had an active role supporting haematopoiesis [16]. Furthermore, experimental evidence has demonstrated the interaction between stroma and haematologic cancer cells (HCCs) [17] (Figure 1). This is especially relevant in the case of HCSCs that house in niches contributing to a pro-tumourigenic environment [18, 19]. Therefore, the key challenges of HMs are to decipher in detail who participates and how in these complex networks that synergize to promote cancer progression.

## HYPOXIA IN HMS AND THE STROMAL COMPARTMENT

Hypoxia is, by definition, a state of reduced oxygenation that influences biological functions [20]. Since the initial indication made by Thomlinson and Gray, it is well established that hypoxia is a characteristic feature of solid tumours [21]. Unlike the normal tissue vasculature, the primitive and chaotic tumour neovasculature is unable to meet all the oxygen and nutrients demands. Nevertheless, this poor and hostile milieu drives cancer cell survival, cancer stem cell maintenance, metabolic reprogramming, angiogenesis and modulation of immune response, so that tumour hypoxia is associated with aggressiveness, metastatic spread and relapse [22].

Compared to most healthy tissues, the bone marrow (BM) environment is characterized by low oxygen availability. A relatively low level of oxygen is indeed a hallmark of the BM stem cell niche, and hypoxia induces the secretion of several growth factors and cytokines such as SDF-1/CXCL12 (stromal cell-derived factor), VEGF (vascular endothelial growth factor) and interleukin-6 involved in haematopoietic stem cells (HSCs) maintenance [23–27]. *In vitro* studies have shown that myelomatous BM environment is more hypoxic than the normal BM [28]. Jensen *et al*. remarked an increase in BM hypoxia during disease progression using a rat AML model [29]. Moreover, using pimonidazole staining for measuring hypoxia levels, Konopleva et al. elegantly demonstrated the high prevalence of hypoxia in human leukaemic BM [30]. Therefore, hypoxia certainly affects the different components of BM and modulates the highly complex and strictly regulated interactions between cancer and stromal cells. Hypoxia sustained AML- and CML-initiating cell maintenance, although contradictory effects on HCCs proliferation have been reported [29, 31–39]. In addition, neoangiogenesis and increased release of hypoxia-induced angiogenic cytokines such as VEGF, bFGF (basic-fibroblast growth factor) and angiopoietin 1 & 2 have been reported in AML and childhood ALL patients [40–42]. I*n vitro* and *in vivo* studies have shown that angiogenic factors favour MM tumour development and lymphoma progression [43–53]. Hypoxia induces metabolic changes, enhances survival, reduces differentiation and promotes self-renewal of mesenchymal/stromal cells [54–56]. Furthermore, co-culture with these cells in hypoxia promotes maintenance and expansion of normal HSCs and human AML cells [39, 56, 57]. Finally, the poorly oxygenated niche and the hypoxia-induced glycololytic metabolism have been linked to chemoresistance in B-ALL, T-ALL, AML, lymphoma and MM cases [58–76].

## HIF, THE MASTER HYPOXIA-SIGNALLING MEDIATOR: IMPLICATIONS IN HMS AND THE STROMAL COMPARTMENT

The hypoxia-inducible transcription factors (HIFs) are central regulators of the cellular response to hypoxia [77]. HIF is a heterodimer composed of one of three oxygen-regulated HIF-α subunits (HIF-1α, HIF-2α and HIF-3α) and the constitutively expressed HIF-β subunit [78, 79]. HIF-1α and HIF-2α, also known as endothelial PAS protein (EPAS1), are the major activators of hypoxia-induced gene transcription, but, to date, little is known about expression and function of HIF-3α [80]. HIF-α proteins share similar structural domains such as an N-terminal basic helix-loop-helix (bHLH) domain involved in DNA binding, two Per-ARNT-Sim (PAS) domains allowing dimerisation, an oxygen-dependent degradation domain (ODDD), and the transactivation domain (TAD). While HIF-3α contains only one TAD, HIF-1α and HIF-2α contain an N-terminal (NTAD) and a C-terminal (CTAD) transactivation domain for recruitment of transcriptional coactivators [81]. In well-oxygenated cells, HIF-α subunits are hydroxylated by the family of prolyl hydroxylase domain-containing proteins (PHDs) on two conserved proline residues (Pro402 and Pro564 in the Human HIF-1α sequence) within the ODDD [82]. The hydroxylated motif allows the binding of the von Hippel-Lindau (VHL) protein, which mediates HIF-α ubiquitination and the further targeting to the proteasome for degradation [83]. While the PHD family consists of four PHDs, PHD1, 2 and 3 have been characterized much more extensively than PHD4, which is bound to the membrane of the reticulum endoplasmic [84–86]. Moreover, PHD2 has been described to be the main PHD controlling HIF-1α stability and levels in normoxia [87]. PHDs act as intracellular molecular sensors that use O_2_ as a substrate, and thus, their activity is compromised upon hypoxia [88, 89]. Thereby, HIF-α evades PHD/pVHL-mediated degradation, dimerises with HIF-1β and the HIF complex binds to specific HIF-response elements (HRE) of target genes. The oxygen-dependent hydroxylation of an asparagine residue in the CTAD of HIF-α (Asn803 in the Human HIF-1α sequence) by Factor Inhibiting HIF (FIH) negatively regulates HIF-target gene expression by impairing the recruitment of the co-activators CBP/p300 [90].

In addition to hypoxia, a number of studies have reported HIF-α sustained protein expression independently of oxygen availability. Growth factors and cytokines such as EGF (epidermal growth factor), FGF-2, heregulin, insulin, IGF1&2 (insulin-like growth factor 1 and 2), IL-1β, TNF-α (tumour necrosis factor α) and factors specifically involved in haematopoiesis such as SCF (stem cell factor) and thrombopoietin positively regulate HIF activity [91–98]. In addition to loss of function mutations in von Hippel-Lindau (VHL) and PTEN, or gain of function mutations in Myc, Ras and Raf [99–102], prevalent mutations found in HMs also promote HIF expression and activity. Hence, activating mutations of FLT3 (Fms-like tyrosine kinase 3), recognized as the most common molecular abnormality in AML [103], increase HIF-α accumulation via the PI3K/AKT/mTOR pathway [104, 105]. Bcr/Abl, an oncoprotein present in most CML cases but also found in ALL and AML patients, induces HIF-1α similarly to FLT3 [106]. Src, another proto-oncogen with a relevant role in HMs, activates HIF through the NADPH oxidase/Rac pathway [107]. NPM (nucleophosmin or nucleolar phosphoprotein B23), which is mutated and chromosomally translocated in many HMs, stimulates HIF activity by inactivating p14ARF [108, 109]. Moreover, contradictory results have been observed regarding IDH mutations and HIF-α accumulation [110–112].

**Figure 2:**
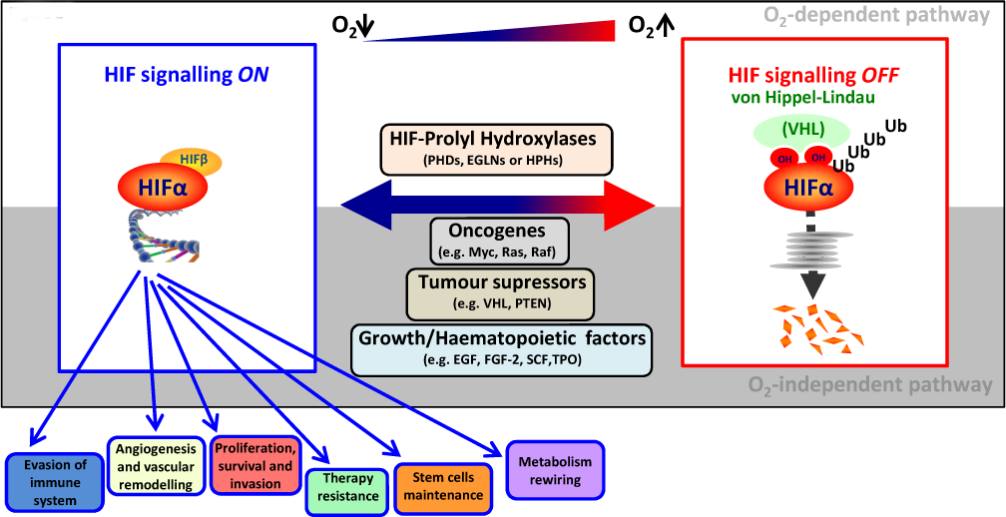
O_2_-dependent and -independent HIF signalling pathway In well-oxygenated cells, the hydroxylation of HIF-α catalyzed by PHDs triggers VHL-mediated ubiquitination and subsequent degradation into the proteasome. In contrast, low oxygen availability stabilizes HIF-α by compromising PHD activity. Moreover, activation of oncogenes, loss of tumour suppressors, growth factors, specific prevalent mutations found in HMs and factors involved in haematopoiesis are also able to upregulate HIF-α independently of oxygen availability. Once stabilized, HIF-α translocates to the nucleus, binds to HIF-β and regulates the expression of genes promoting tumour progression.

HIF drives the transcription of genes involved in many pathways promoting angiogenesis and vascular remodelling, proliferation, survival and invasion of cancer cells and stem cell maintenance [113] (Figure 2). Overall, increased HIF-α expression is correlated with tumour growth and therapy resistance and, therefore, with disease relapse [114, 115]. Accordingly, sustained expression of HIF-α is a marker of poor prognosis not only in several types of solid tumours but also in HMs [116–129]. Therefore, inhibition of HIF-α (either by RNAi or small molecules) resulted in a failure of primary cells to form *in vitro* colonies and significantly increased disease-free survival *in vivo* [36, 118, 130–136]. However, the specific role of HIFs in HMs seems to be controversial and contradictory data have also been published. Thus HIF-1α has been reported to induce cell differentiation in AML, and loss of HIF-1α resulted in faster development of the disease and reduced survival [137–142]. Similarly, HIF-1α overexpression associated with increased survival in patients with diffuse large B-cell lymphoma [143]. Such an apparent controversy could be explained by the different system used (mouse versus human), the unspecificity of shRNA/drugs compared to knock-out models or the hypoxic exposure. Therefore, further studies will be certainly needed to clarify the oncogenic and/or tumour suppressor activity of HIF signalling in HMs, and particularly within HCSCs.

Homing and subsequent adhesion of cancer stem cells to the vascular and the endosteal niche triggers the *in vivo* tumour-stroma interactions. The chemokine receptor CXCR4 (C-X-C chemokine receptor type 4) and its ligand SDF-1/CXCL12 mediate this process and it is well known that HIF-1α regulates the expression of both [144, 145]. Moreover, the involvement of this axis in chemoresistance has been deeply demonstrated in HMs [146–151]. In this regard, it has been recently published that HIF-1α increases the interaction of CLL cells with the stroma and that stromal cells protect mantle cell lymphoma cells from the cytotoxic effect of chemotherapeutic agents [152, 153]. Furthermore, aberrant expression of HIF-1α in bone marrow endothelial cells has been linked to drug resistance and recurrence in patients with MM [126].

## HYPOXIA AND/OR HIFS AS TARGETS TO TREAT HMS

All the previously reported data emphasize the relevance of fighting against hypoxia and HIF signalling in HMs. Not only HCCs but also the stromal compartment should be targeted to fully frustrate the pro-tumourigenic environment promoted by their interaction. The therapeutic strategy aiming to directly target hypoxic cells within tumours remains a challenging approach, while success in clinical trials has so far proved elusive [154]. This approach relies mostly on the use of hypoxia-activated prodrugs (HAPs) or bioreductive drugs. The prodrugs are inactive medications that require metabolization before exhibiting pharmacological effects. In particular, HAPs require activation by oxygen-inhibited enzymes (typically by 1 or 2 electron oxidoreductases) to generate cytotoxic compounds [154]. One of the most extensively examined HAPs is evofosfamide (TH-302), the reductive activation of which generates bromo-isophosphoramide mustard (Br-IMP), a potent alkylating agent. TH-302 exhibited specific hypoxia-dependent cytotoxicity when tested in primary ALL and AML samples *in vitro* and reduced the AML stem cell pool *in vivo* [155, 156]. Similarly, TH-302 induced cell cycle arrest and triggered apoptosis in severely hypoxic conditions in MM, while had no effect at similar doses in normoxic conditions [38]. A phase I/II clinical trial in relapsed or refractory ALL or AML has been carried out using PR-104, which also results in the generation of a DNA-damaging metabolite. In this study, PR-104 demonstrated measurable clinical activity but also significant toxicity at the doses administered in the trial [30]. It is worth nothing that novel HAPs designed to release targeted therapeutics (pioneered by TH-4000 that releases EGFR tyrosine kinase inhibitor) have been recently developed, though to our knowledge no data related to HMs are available [154].

Regarding the use of drugs directly targeting HIF, several chemical inhibitors have been tested in different models. Echinomycin (NSC-13502) is an antibiotic derivative from the quinoxaline family, which inhibits HIF-1α/DNA binding activity. This inhibitor has been previously evaluated in clinical trials in solid tumours, though with disappointing results [157]. Interestingly, echinomycin abrogated *in vitro* and *in vivo* lymphoma and AML growth through preferential targeting of HCSCs [132, 133, 158]. HIF-1α inhibition by 2-methoxyestradiol (2ME2), and endogenous metabolite of oestrogen that disrupt microtubule architecture, and YC-1(3-(5′hydroxymethyl-2′-furyl)-1-benzy-lindazole) induced cell death in different HMs [159]. L-ascorbic acid was also able to specifically inhibit the proliferation of human CML cells via downregulation of HIF-1α transcription [135]. EZN-2968, a small 3^rd^ generation antisense oligonucleotide against *HIF1A* mRNA, delayed acute promyelocytic leukaemia (APL) and MM progression [160, 161]. EZN-2968 has also been reported to block the interaction between MM cells and BM stromal cells through HIF-1α inhibition [161]. Furthermore, the combination of EZN-2088, a polyethylene glycol conjugate of irinotecan (PEG-SN38), with all-trans retinoic acid (ATRA) synergized to eradicate preclinical models of PML-RARα (promyelocytic leukaemia protein-retinoic receptor antagonist alpha) and PLZF (promyelocytic leukaemia zinc finger)-RARα-driven leukaemia [160]. More recently, it has been shown that chetomin, a small molecule able to disrupt HIF-1α binding to the p300 coactivator, exhibited antitumor activity in primary MM cells from patients [162]. Similarly, acriflavine, another FDA-approved HIF inhibitor, has demonstrated specificity towards CML stem cells [163]. Moreover, it should be pointed out the relevance of bortezomib (PS-341), a proteasome inhibitor, in the treatment of MM and mantle cell lymphoma patients. Relapse/refractory but also newly diagnosed cases benefit of bortezomib either as single agent or combined with other therapies [164, 165]. Indeed, bortezomib has been reported to repress HIF-1 (and not HIF-2)-dependent transcriptional activity by reinforcing FIH-mediated inhibition of p300 recruitment [166]. Supporting combinatorial therapeutic options, TH-302 together with bortezomib induced MM cell cycle arrest and triggered apoptosis in severe hypoxic conditions, while having no effect at similar doses in normoxic conditions [167]. More recently, PT2385 has been developed as a selective agent that blocks HIF-2α with potent anti-cancer activity in preclinical models of advanced clear cell renal cell carcinoma (ccRCC) [168–170]. However, this inhibitor has not been still tested for the treatment of HMs. Finally, it is worth mentioning that several agents in current clinical practice to treat HMs directly inhibit HIF-α, which might contribute to their therapeutic efficacy. This is indeed the case for imatinib or the topoisomerase I inhibitor, topotecan. Furthermore, the potential impact of rituximab on HIF1α expression levels might argue its favorable prognostic value in patients with DLBCL treated with this monoclonal antibody [123].

## CONCLUSION

Data reported from several research laboratories claim that hypoxia and HIF-mediated signalling favour haematologic and lymphoid tumour progression and relapse. The results using hypoxia-activated prodrugs and HIF-α inhibitors in different preclinical and clinical models are really promising. Hence, these data give exciting perspectives to define new and better therapeutic approaches that may benefit patients suffering from HMs.
